# Supplementary material for: Characterization of Organosolv Lignins and Their Application in the Preparation of Aerogels
Source: Materials (Basel). 2022 Apr 13;15(8):2861. doi: 10.3390/ma15082861 (PMC9029481; doi:10.3390/ma15082861)
Supplement: Supplementary file 1 [file materials-15-02861-s001.zip › materials-1672128-supplementary.pdf]

materials

Supplementary Materials

Characterization of Organosolv Lignins and Their Application in the Preparation of Aerogels

Piia Jõul [1,†], Tran T. Ho [1,†], Urve Kallavus [2], Alar Konist [3], Kristiina Leiman [1], Olivia-Stella Salm [1], Maria Kulp [1], Mihkel Koel [1] and Tiit Lukk [1,*]

[1] Department of Chemistry and Biotechnology, Tallinn University of Technology, Akadeemia tee 15, 12618 Tallinn, Estonia; piia.joul@gmail.com (P.J.); thihol@taltech.ee (T.T.H.); kristiina.leiman@taltech.ee (K.L.); olivia-stella.salm@taltech.ee (O.-S.S.); maria.kulp@taltech.ee (M.K.); mihkel.koel@taltech.ee (M.K.)

[2] Department of Mechanical and Industrial Engineering, Tallinn University of Technology, Ehitajate tee 5, 19086 Tallinn, Estonia; urve.kallavus@taltech.ee

[3] Department of Energy Technology, Tallinn University of Technology, Ehitajate tee 5, 19086 Tallinn, Estonia; alar.konist@taltech.ee

* Correspondence: tiit.lukk@taltech.ee

† These authors contributed equally to this work.

Table S1. The detailed HSQC assignment of six organosolv lignin samples.

Labels	δC/δH (ppm)	Assigment
**Hk_{α}**	44.66/3.79	Cα-Hα in Hibbert's ketone structures (Hk)
**C_{β}**	52.89/3.45	Cβ-Hβ in phenylcoumaran substructures (C)
**B_{β}**	53.45/3.05	Cβ-Hβ in resinol substructures (B)
**E_{β}**	53.62/2.83	Cβ-Hβ in β-1' linkage (E)
**OMe**	55.55/3.73	C-H in methoxy
**I_{γ}**	59.33/4.03	Cγ-Hγ in cinamyl alcohol end-groups (I)
**A/A'_{γ}**	59.65/3.59; 3.25	Cγ-Hγ in β-O-4' substructures (A/A')
**C_{γ}**	62.72/3.65	Cγ-Hγ in phenylcoumaran substructures (C)
**A'_{α_OEt}**	63.60/3.32	Ethoxylated Cα-Hα in β-O-4' substructures (A')
**Hk_{γ}**	66.91/4.16	Cγ-Hγ in Hibbert's ketone structures (Hk)
**B_{γ}**	70.94/4.18; 3.82	Cγ-Hγ in resinol substructures (B)
**$A_{\alpha(G)}$**	71.18/4.87	Cα-Hα in β-O-4' linked to G unit (A)
**$A_{\alpha(S)}$**	71.90/4.86	Cα-Hα in β-O-4' linked to S units (A)
**A'_{α}**	79.72/4.45 and 81.01/4.53	Cα-Hα in β-O-4' substructures (A')
**$A'_{\beta(G)}$**	82.06/4.38	Cβ-Hβ in β-O-4' linked to G unit (A')
**$A_{\beta(G)}$**	83.10/4.28	Cβ-Hβ in β-O-4' linked to G unit (A)
**$A'_{\beta(S)}$**	84.55/4.19	Cβ-Hβ in β-O-4' linked to S unit (A')
**B_{α}**	84.80/4.66	Cα-Hα in resinol substructures (B)
**$A_{\beta(S)}$**	85.76/4.12	Cβ-Hβ in β-O-4' linked to S unit (A)
**C_{α}**	86.73/5.45	Cα-Hα in phenylcoumaran substructures (C)
**T_{8}**	94.14/6.58	C8-H8 in tricin substrcutures (T)
**T_{6}**	98.82/6.23	C6-H6 in tricin substrcutures (T)
**$S_{2,6}$**	104.30/6.60	C2,6-H2,6 in syringyl units (S)
**$T_{2',6'}$**	103.97/7.32	C2',6'-H2','6 in tricin substrcutures (T)
**T_{3}**	104.62/7.05	C3-H3 in tricin substrcutures (T)
**$S'_{2,6}$**	106.31/7.30	C2,6-H2,6 in oxidized (Cα=O) syringyl units (S')
**$S_{2,6\text{-}LBHK}$**	106.67/6.53	C2,6-H2,6 in syringyl unit bound Hibbert's ketone (S-LBHK)

**P$_2$**	109.99/7.26	C2-H2 in methyl-substituted phenylcoumarone substructures (P)
**G$_2$**	110.10/6.92	C2-H2 in guaiacyl units (G)
**Fer$_2$**	111.06/7.31	C2-H2 in ferulate (Fer)
**G$_{5\text{-LBHK}}$**	112.89/6.90	C5-H5 in guaiacyl unit bound Hibbert's ketone (G-LBHK)
**G$_{2\text{-LBHK}}$**	113.31/6.84	C2-H2 in guaiacyl unit bound Hibbert's ketone (G-LBHK)
**H$_{3,5}$**	114.61/6.72	C3,5-H3,5 in p-hydroxylphenyl (H)
**G$_5$**	114.93/6.93; 6.79	C5-H5 in guaiacyl units (G)
**PCA$_{3,5}$**	115.58/6.79	C3,5-H3,5 in p-coumarate (pCA)
**G$_6$**	118.32/6.76	C6-H6 in guaiacyl units (G)
**P$_6$**	119.53/7.21	C6-H6 in methyl-substituted phenylcoumarone substructures (P)
**G$_{6\text{-LBHK}}$**	121.36/6.64	C6-H6 in guaiacyl unit bound Hibbert's ketone (G-LBHK)
**Fer$_6$**	122.35/7.15	C6-H6 in ferulate (Fer)
**H$_{2,6}$**	127.82/7.21	C2,6-H2,6 in p-hydroxylphenyl (H)
**PCA$_{2,6}$**	130.08/7.54	C2,6-H2,6 in p-coumarate (PCA)
**PB$_{2,6}$**	131.13/7.69	C2,6-H2,6 in p-benzoate (Pb)
**PCA$_\alpha$/Fer$_\alpha$**	144.42/7.53	Cα-Hα in p-coumarate (PCA) and ferulate (Fer)

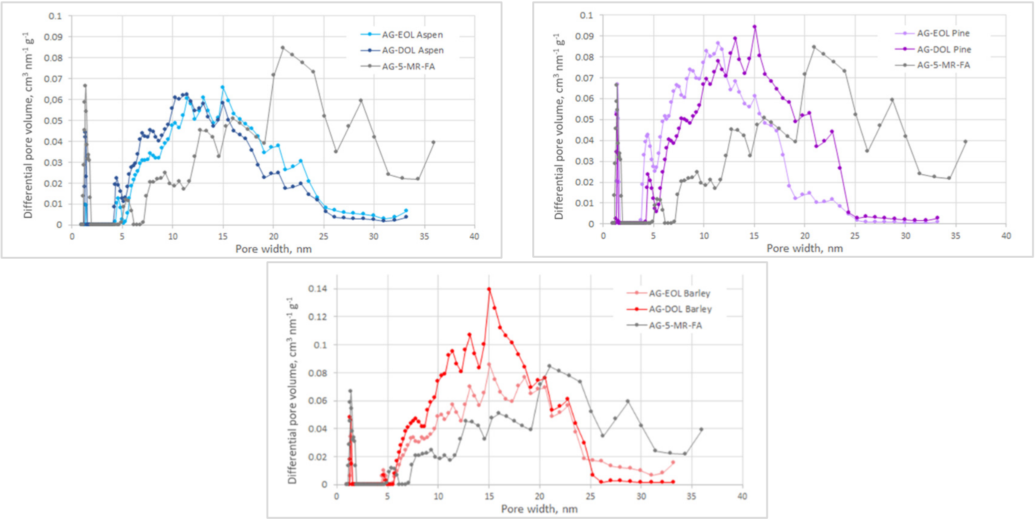

Figure S1. Pore-size distributions of lignin-based (75% of the 5-MR was replaced by lignin) and 5-MR-FA aerogels.
